# The PE-PPE Domain in Mycobacterium Reveals a Serine α/β Hydrolase Fold and Function: An *In-Silico* Analysis

**DOI:** 10.1371/journal.pone.0016745

**Published:** 2011-02-10

**Authors:** Rafiya Sultana, Karunakar Tanneeru, Lalitha Guruprasad

**Affiliations:** School of Chemistry, University of Hyderabad, Hyderabad, India; St. Petersburg Pasteur Institute, Russian Federation

## Abstract

The PE and PPE proteins first reported in the genome sequence of *Mycobacterium tuberculosis* strain H37Rv are now identified in all mycobacterial species. The PE-PPE domain (Pfam ID: PF08237) is a 225 amino acid residue conserved region located towards the C-terminus of some PE and PPE proteins and hypothetical proteins. Our *in-silico* sequence analysis revealed that this domain is present in all Mycobacteria, some *Rhodococcus* and *Nocardia farcinica* genomes. This domain comprises a pentapeptide sequence motif GxSxG/S at the N-terminus and conserved amino acid residues Ser, Asp and His that constitute a catalytic triad characteristic of lipase, esterase and cutinase activity. The fold prediction and comparative modeling of the 3-D structure of the PE-PPE domain revealed a “serine α/β hydrolase” structure with a central β-sheet flanked by α-helices on either side. The structure comprises a lid insertion with a closed structure conformation and has a solvent inaccessible active site. The oxyanion hole that stabilizes the negative charge on the tetrahedral intermediate has been identified. Our findings add to the growing list of serine hydrolases in mycobacterium, which are essential for the maintenance of their impermeable cell wall and virulence. These results provide the directions for the design of experiments to establish the function of PE and PPE proteins.

## Introduction

The complete sequence of the *M. tuberculosis* genome in the year 1998 provided vital information regarding the genes, physiology and pathogenesis responsible for tuberculosis (TB). An important finding from this genome sequence was the identification of PE and PPE gene families that comprise about 10% of the total genome [1]. The *M. tuberculosis* strain H37Rv genome comprises 167 PE and PPE proteins. Subsequently, it was observed that these two gene families are mycobacteria specific. The PE protein family is characterized by the presence of 110 amino acid N-terminal domain with a PE (Pro-Glu) sequence motif at positions 9 and 10. The PPE protein family is characterized by the presence of 180 amino acid N-terminal domain with a PPE (Pro-Pro-Glu) sequence motif at positions 9, 10 and 11. Nearly 50% of these proteins comprise only the characteristic N-terminal conserved domain, while the other members comprise C-terminal extensions. Based on the composition of the C-terminal extensions, the PE and PPE proteins were further classified into various subfamilies [2]. These variable C-terminal extensions form a source of antigenic variation among different strains of this bacterium that lead to a speculation that these protein families could be immunologically important.

Despite the availability of the sequence information of the TB genome for over 12 years, identification of the precise function of all the PE and PPE proteins has been limited and is an important area for both basic and applied research aimed at the diagnosis and therapy of TB. The PE and PPE proteins are cell wall associated and surface exposed [2]–[5]. It has been shown that PE and PPE genes are not randomly distributed in the genome but clusters of PE and PPE genes are present in operons and that they co-transcribe as pairs of PE and PPE proteins [6], [7]. For example, the operon containing PE25 and PPE41 genes co-transcribe and their products interact with each other. The 3-D structure of the heterodimer complex of PE25 and PPE41 has shown that the PE and PPE domains contribute two α-helices each to form a four helical bundle at the heterodimer interface [8]. The PE25-PPE41 protein complex has been shown to induce increased humoral and cell mediated immune response [9]. Further, differential immunological response to the PE25-PPE41 complex versus the individual proteins was reported. Three PE family members Rv1169c, Rv0978c and Rv1818c have been shown to display a strong but differential B-cell humoral response among different clinical categories of TB patients indicating the possibility of differential utility in the serodiagnosis of TB [10]. The PPE protein Rv2430c has been reported to induce a strong B-cell response, pointing to the immunodominant nature of the protein [11].

The enzymatic functional role of PE and PPE proteins has not been reported so far with the exception of Rv3097c. The C-terminal domain of Rv3097c, a PE_PGRS63 protein is homologous to the hormone-sensitive lipase family and is characterized by the presence of conserved GDSAG motif and exhibits triacylglycerol hydrolase activity [12]. In our earlier studies, we reported a 225 amino acid residue conserved domain in some PE, PPE and hypothetical proteins in mycobacterial species [2]. We termed it the PE-PPE domain (Pfam ID: PF08237) because it was commonly observed towards the C-terminus of some PE and PPE proteins. In this work, using computational approaches, we identified that the PE-PPE domain has a serine α/β hydrolase fold and function and specifically possess esterase, lipase or cutinase activity. Cutinases are serine hydrolases which cleave cutin, a complex glycolipid polymer consisting of hydroxy and epoxy fatty acids. Some cutinases from *Streptomyces scabies* [13], *Pseudomonas putida* [14], [15] and *Thermobifida fusca* [16], [17] have been characterized. Lipases are lipolytic hydrolases that catalyze the hydrolysis of carboxyl ester bonds in water insoluble mono-, di- and triglycerides to liberate fatty acids and alcohols in aqueous solutions. Examples of bacterial lipases are from *Staphylococcus xylosus* [18] and *Acinetobacter baumannii* BD5 [19]. While the lipases have the ability to hydrolyse long chain acylglycerols, esterases function by hydrolysing ester substrates with short chain fatty acids. Some examples of bacterial esterases are from *Lactobacillus plantarum* [20] and *Thermus Scotoductus* [21].

In this work, using computational studies, we identified the PE-PPE domain to comprise a serine α/β hydrolase fold and a common serine hydrolase enzymatic function. Also the lid insertion close to the active site and the oxyanion hole has been characterized. Our results provide direction for further experimental design to establish the esterase, lipase or cutinase activity and 3-D structure of the PE-PPE domain.

## Methods

The amino acid sequence regions corresponding to the PE-PPE domain from *M. tuberculosis* strain H37Rv were obtained from the NCBI (http://www.ncbi.nlm.nih.gov/) protein sequence databank. For example, in the protein Rv1430, the region between the amino acid residues 108 to 337 corresponds to the PE-PPE domain. The PE-PPE domain regions were used for sequence analysis and protein 3-D structure modeling. The BLAST (Basic Local Alignment Search Tool) program was used to identify regions of similarity between the query sequence and sequences in the database (DNA or protein). This was followed by the Position Specific Iterative BLAST (PSI-BLAST) searches that use a profile or position specific scoring matrix that is automatically constructed from the multiple sequence alignment of highest scoring hits from the output of BLAST. The profiles are refined during each iteration such that even the weak homologues in the database are identified resulting in increased sensitivity. A PSI-BLAST search [22] of the PE-PPE domain region against non-redundant database at NCBI was carried out to identify all proteins that comprise this domain. The multiple sequence alignment of the PE-PPE domain regions was carried out using CLUSTALW [23] program available at http://www.ebi.ac.uk/Tools/clustalw2/index.html. A multiple sequence alignment allows the study of the extent of conservation and variation in their DNA or protein sequences. The signal peptide server [24] available at http://www.cbs.dtu.dk/services/SignalP/ was used to predict the presence and location of the signal peptide cleavage sites in the hypothetical proteins comprising the PE-PPE domain.

The PSI-BLAST program was also used to search against sequences of known 3-D structure available in the Protein Structure Databank (PDB) available at www.rcsb.org/, in order to select appropriate templates for constructing 3-D structure models of the PE-PPE domain using comparative modeling methods. Further, the fold prediction method FUGUE [25] available at http://tardis.nibio.go.jp/fugue/prfsearch.html was used to identify the probable structural fold for the PE-PPE domain. This method identifies the likely fold of the query sequence and also generates an alignment between the sequences of the query protein and the probable template structures. FUGUE uses the global-local algorithm to align a sequence-structure pair when they greatly differ in length and otherwise uses the global algorithm. This method employs automatic selection of alignment algorithm with detailed structure-dependent gap penalties. The gap penalty at each position of the structure is determined according to its solvent accessibility, its position relative to the secondary structure elements (SSEs) and the conservation of the SSEs. As a result, the alignments generated by FUGUE represent a better relatedness between the amino acid sequences of the query protein and the template structure. We have used these sequence alignments to build the 3-D model structures of the PE-PPE domains using HOMOLOGY module in InsightII (Accelerys Inc, USA) that implements the methodology described in MODELLER [26].

MODELLER is a homology or comparative modeling program for constructing a 3-D model of a protein structure from its amino acid sequence. It is based on the alignment between the sequences to be modeled with the sequence of known template structure. The program automatically constructs a model for all non-hydrogen atoms in the protein structure by the satisfaction of spatial restraints that includes non-homologous loops and energy optimization of the final model. As a result, the proteins built on low sequence homology also yield good quality and reliable models. The models were validated using PROCHECK [27] available at http://nihserver.mbi.ucla.edu/SAVES/ and Verify_3D [28] available at http://nihserver.mbi.ucla.edu/SAVES/. Given the coordinates of the model protein, parameters such as the distribution of phi, psi and chi torsion angles and additional parameters including proline phi angles, peptide bond planarities, disulfide bond lengths can be measured. These parameters were used to evaluate the stereochemical geometry and quality of a protein structure model using the automated method PROCHECK. Since the sequence homology between the PE-PPE domain and known 3-D structures is very low, the validation of the overall fold of the modeled structure is very important. In order to achieve this, the 3-D protein model structure is compared with its own amino acid sequence, using a 3D profile, which is computed from the atomic coordinates of the structure 3D profiles of correct protein structures that match their own sequences with high scores. Verify_3D validates the compatibility of 3-D model structure with its 1-D amino acid sequence.

The probable structural folds identified by the FUGUE method and 3-D structures of the PE-PPE domain constructed were superimposed using MAPSCI server [29] available at http://www.geom-comp.umn.edu/mapsci/. The structural overlay identifies common substructures in a set of proteins that will assess the relatedness between structures and the evolutionary history and function.

## Results

**Figure 1:**
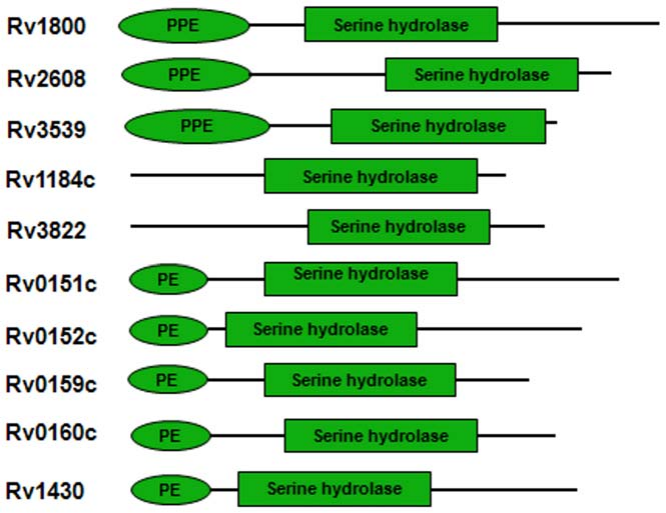
Domain architecture of proteins in *M. tuberculosis* strain H37Rv genome comprising the PE-PPE domain that encodes a serine hydrolase.

The PSI-BLAST searches identified that the PE-PPE domain is present in all mycobacterial genomes. In *M. tuberculosis* H37Rv strain, ten proteins comprise the PE-PPE domain. The domain architecture of these proteins is shown in Figure 1. The detailed genome-wide sequence analysis revealed that homologs of PE and PPE proteins (Rv0151c, Rv0152c, Rv0159c, Rv0160c, Rv1430, Rv1800, Rv2608, Rv3539) are present in *M. tuberculosis*, *M. bovis*, *M. kansasii*, *M. marinum* and *M. ulcerans* genomes. The homologs of hypothetical proteins (Rv1184c and Rv3822) that comprise only the PE-PPE domain are present in *M. tuberculosis, M. bovis*, *M. parascrofulaceum*, *M. smegmatis*, *M. vanbaalenii*, *M. abscessus*, *M. avium*, *M. leprae* and *Mycobacterium* sp. JLS, KMS and MCS. The list of proteins comprising the PE-PPE domain in mycobacterial genomes is shown in Table S1. Further, we also observed that the homologs of the hypothetical proteins are present in *Nocardia farcinica* (YP_120155.1) and some species from *Rhodococcus* (YP_002767666.1, YP_002764179.1, YP_002783136.1, YP_002781243.1, YP_705817.1, ZP_04388076.1, ZP_04387701.1, ADD80824.1) that belong to actinobacteria but these genomes do not comprise the PE and PPE family proteins.

According to the signal peptide server, the hypothetical protein, Rv1184c has its signal peptide cleaved at the N-terminus between Ala-26 and Ser-27 and is likely to be a secreted protein. The hypothetical protein Rv3822 was predicted as a non-secretory protein by the signal peptide server.

The multiple sequence alignment of the PE-PPE domain (Figure S1) provides insights into the relatedness of their amino acid sequences. We observed that the pentapeptide sequence motif GxSxG/S and the amino acid residues Ser-199, Asp-276 and His-302 (numbering according Rv1430) that form the catalytic triad are highly conserved. The second position in the pentapeptide sequence is occupied by a hydrophobic residue such as Leu/Tyr/Trp/Phe with the exception of Rv2608 where it is replaced by Thr. The fourth position is occupied by Gln in all the proteins with the exception of Rv3539 where it is replaced by Met.

The PSI-BLAST search against the PDB identified the PDB_ID: 1G29 with E- value 0.068. This PDB_ID corresponds to the structure of ATPase subunit of the trehalose/maltose ABC transporter of *Thermococcus litoralis*. The high E- value is indicative of very low sequence homology between the PE-PPE domain and the identified 3-D structure. However, FUGUE program identified PDB_ID: 3AJA as the probable fold with highest Z- score of 21.62. Other proteins identified as probable structures were PDB_IDs: 1CEX, 1BS9, 2CZQ and 3HC7 with Z- scores >6.0 indicating the confidence in fold prediction results. The PDB_ID: 3AJA corresponds to a lipase from *M. smegmatis* strain MC2155, the PDB_ID: 1CEX corresponds to the crystal structure of *Fusarium solani* cutinase, the PDB_ID: 1BS9 corresponds to an acetylxylan esterase from *Penicillium purpurogenum*, the PDB_ID: 2CZQ corresponds to a cutinase like protein from *Cryptococcus* sp. S-2, the PDB_ID: 3HC7 corresponds to a mycobacteriophage esterase that cleaves the mycolylarabinogalactan bond to release free mycolic acids.

**Figure 2:**
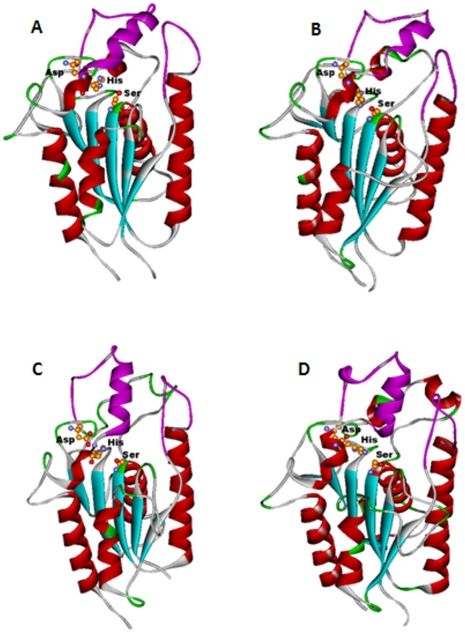
The overall fold of the serine hydrolases. (A) Rv1430 PE-PPE domain. (B) Rv1800 PE-PPE domain. (C) Rv1184c PE-PPE domain. (D) PDB_ID: 3AJA used as template for homology modeling. The helices are represented in red, strands in blue, the lid insertion in pink. The side chains of the amino acids in the catalytic triad are indicated in ball and stick.

**Figure 3:**
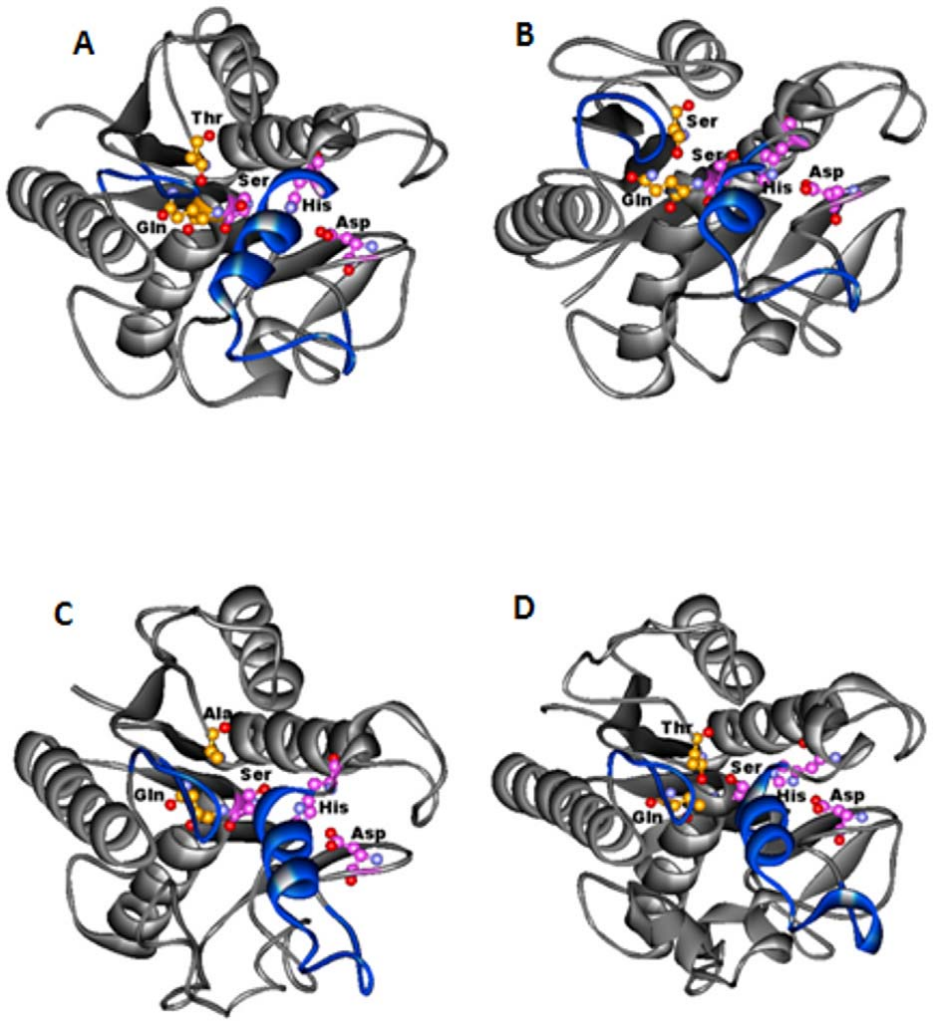
The top view of the serine hydrolases indicating a closed conformation of the lid insertion on the active site. (A) Rv1430 PE-PPE domain. (B) Rv1800 PE-PPE domain. (C) Rv1184c PE-PPE domain. (D) PDB_ID: 3AJA. The protein is represented in grey, the lid insertion in blue, the side chains of the amino acids in the catalytic triad and oxyanion hole are indicated in ball and stick.

The sequence alignment generated between the H37Rv query proteins and the PDB_ID: 3AJA by the FUGUE method was used for the 3-D structure modeling of the PE-PPE domain using MODELLER. All models exhibited an overall α/β hydrolase fold with central β-sheet, flanked by α-helices on either side of the sheet (Figure 2). The location of the pentapeptide sequence motif and the catalytic amino acid residues Ser, Asp and His are highly conserved in all the structures. A close observation of the region around the active site indicated the presence of the lid insertion region and solvent inaccessible catalytic Ser indicating the closed conformation adopted by these hydrolase structures (Figure 3). Most of the classical lipases (for example PDB_IDs: 2Z8X, 5TGL, 2VEO, 4TGL) possess a lid insertion region over the active site that keeps the active site in a closed conformation. These lids are displaced during interfacial activation allowing the substrate to be activated by opening up and exposing the catalytic triad. Another interesting aspect of the serine hydrolases is the presence of an oxyanion hole that constitutes a part of the active site. The oxyanion hole is formed by the amino acid residues whose main chain nitrogen atoms act as hydrogen donors to the hydrolysed substrate, stabilizing the negative charge on the tetrahedral intermediate arising from the nucleophilic attack of the catalytic Ser during activation [30]. In bacterial lipases, (for example PDB_IDs: 1IVN, 1CRL) the location of an oxyanion hole has been characterized. Based on the homology between these crystal structures and the constructed structure models of PE-PPE domain, we have identified the location of the oxyanion hole in all our modeled structures. For example, in the case of Rv1430, the residues Gln-200 and Thr-121 are responsible for the formation of the oxyanion hole as indicated in Figure 3.

**Figure 4:**
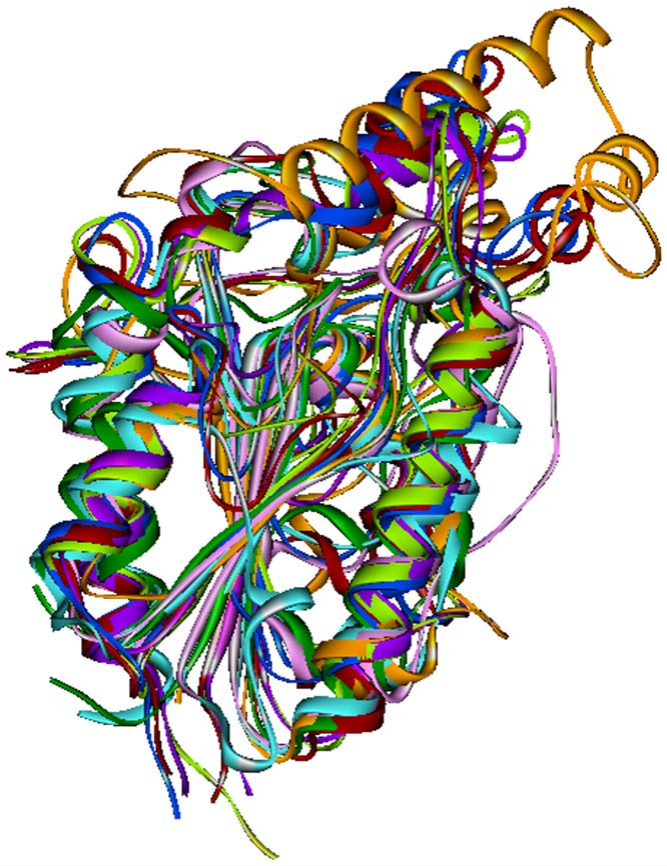
The superposition of the crystal structures of bacterial serine hydrolases and the 3-D structure models of the PE-PPE domain that encode serine hydrolases. PDB_ID: 1BS9 is represented in pink, PDB_ID: 1CEX in cyan, PDB_ID: 2CZQ in green, PDB_ID: 3AJA in red, PDB_ID: 3HC7 in orange, Rv1184c in blue, Rv1430 in purple and Rv1800 in pale green. Only three PE-PPE homology model structures were included for the sake of clarity in structure superposition.

The 3-D structures of the PE-PPE models and the probable structures identified by FUGUE method were superimposed using MAPSCI. These structures superimpose (Figure 4) with 0.8 Å root mean square deviation (RMSD) value indicating structural similarity despite high divergence in the amino acid sequences. The low sequence similarity is also indicated in the corresponding multiple sequence alignment shown in Figure S1.

For the preliminary assessment of the 3-D model structures, the location of amino acid residues in the catalytic triad was examined in order to validate the PE-PPE domain active site. All the protein models constructed satisfied the position of the catalytic triad and are similar to the crystal structures of other lipases. Further, structure assessment using PROCHECK indicated that greater than 85% residues were located in the allowed region of Ramachandran plot [31] and less than 2% residues were located in the disallowed region indicating that the geometrical quality of PE-PPE models was satisfactory. Verify_3D showed that the overall scores of the modeled structures were greater than 85 indicating a good compatibility between the 3-D modeled structure and its corresponding 1-D amino acid sequence. The 3-D structures of some representative models are shown in Figure 2.

Our results indicated that the PE-PPE domain has serine α/β hydrolase fold with a correctly positioned catalytic triad. Further, these structures are highly similar to other bacterial cutinases, esterases and lipases despite low sequence homology. These structures have a lid insertion with closed conformation and the oxyanion hole has been identified.

## Discussion

In *M. tuberculosis* strain H37Rv genome, the PE-PPE domain is present in ten proteins as shown in Figure 1. The detailed genome-wide sequence analysis indicated that the PE-PPE domain comprising proteins are present in all mycobacterial genomes. In addition, the PE-PPE domain is also present in hypothetical proteins of some actinobacteria.

As shown in Figure 1, the presence of this domain towards the C-terminus of some PE and PPE proteins is an example of gene fusion. The occurrence of two genes (A and B) with independent functions, as a single bifunctional gene (A–B) in orthologs or paralogs is an example of gene fusion during evolution. The gene fusion is indicative of the relatedness in their functions [32]. In the context of *M. tuberculosis* strain H37Rv, the fusion of PE and PE-PPE domains in some PE family proteins; or PPE and PE-PPE domains in some PPE family proteins indicated that the serine hydrolase activity of PE-PPE domain is located on the cell surface and one of the roles of the N-terminal PE or PPE domains would be to translocate the serine hydrolase PE-PPE domain to the cell surface.

Further, the presence of ten distinct genes comprising the PE-PPE domain as paralogs in *M. tuberculosis* strain H37Rv is an example of gene duplication. During the evolution of an organism, in order to survive under the selective pressure of the host or environment, the preexisting genes undergo duplication followed by mutations and rearrangements to cater to the new requirements instead of the synthesis of new genes. Therefore, we believe that these ten genes would have closely related hydrolase function with distinct activities. As indicated in Table S1, the distribution of PE-PPE domains in proteins from various mycobacterial genomes is not uniform and these differences could be responsible for the differences in pathogenesis and virulence of the organism and the nature of host.

According to the signal peptide server, Rv1184c is likely to be an exported protein, while Rv3822 is a non-secretory protein suggesting that it could be cell wall associated. However, it has been reported that Culp6 is essentially a cell wall associated protein despite possessing a predicted signal peptide [33] indicating that experimental studies are required to decipher the localization of these hypothetical proteins in *M. tuberculosis*.

The BLAST searches against PDB did not yield useful template for homology based structure modeling. However, the FUGUE method identified the 3-D structures of cutinase, esterase and lipase as probable folds of the PE-PPE domain. These structures are members of serine hydrolase superfamily that have evolved from a common ancestor. The structure models of the PE-PPE domains constructed using MODELLER adopts a conserved 3-D fold with central β-sheet and α-helices on either side. This fold is characteristic of serine α/β hydrolase architecture. The highly conserved catalytic residues form the triad and are suitably positioned to facilitate the hydrolysis activity. Structure analysis identified the lid insertion and oxyanion hole that regulate the enzyme activity. We describe the PE-PPE domain to encode serine α/β hydrolase proteins based on; 1) the pentapeptide sequence motif GxSxG/S, 2) the presence of catalytic triad Ser, Asp and His in the active site, 3) identification of cutinase, lipase and esterase structures from the fold prediction method and 4) the homology models of the PE-PPE domain adopt a serine α/β hydrolase fold. The superimposition of the Cα- trace of all PE-PPE model structures and the crystal structures of bacterial lipases, cutinases and esterases showed a low RMSD suggesting that these domain regions are very similar to the serine hydrolases from other bacterial origin despite very low sequence homology.

In *M. tuberculosis* genome, 250 enzymes were reported to be important for lipid metabolism and 94 members were predicted to contain the α/β- hydrolase fold characteristic of lipases/esterases [34]. Recent review by Singh et al., 2010 [35] provides an overview of lipases and related family members in the virulence of *M. tuberculosis*. In *M. tuberculosis*, the well characterized members of serine hydrolases are three enzymes of antigen 85 complex (Rv3804c, Rv1886c and Rv0129c) that are essential for the transfer of mycolic acid during cell wall biosynthesis. Rv2422c, a serine hydrolase has been identified to be cell wall associated virulence factor [36] or in triacylglycerol utilization during nutrition limiting conditions [37]. In *M. tuberculosis* strain H37Rv, seven cutinase-like proteins (Rv1984c, Rv2301, Rv3451, Rv3452, Rv1758, Rv3802c, Rv3724) were identified based on the bioinformatics analysis [38]. Diverse physiological functions and immunological responses of these cutinases have been reported [32], [39].

A fundamental and important question that needs to be addressed here is the presence of diverse lipid hydrolyzing proteins in *Mycobacterium*. One third of the world population is infected with *M. tuberculosis* and is carried in dormant form. Before entering the dormant form, the bacterium degrades the cell membrane of the host and accumulates the lipids in order to resynthesize complex lipid molecules to suit their survival in the host environment [40]. During dormancy, mycobacteria utilize fatty acid as a source of energy and it has been suggested that *M. tuberculosis* stores fatty acids as triacylglycerol [41]. Microscopic examination has shown that the mycobacteria have intact cell envelope and large lipid inclusion bodies in the cytoplasm [42]. Due to the decrease in the host immunity, the pathogenic bacterium enters the reactivation phase, when the lipid inclusion bodies are hydrolysed and the infection process begins with the onset of disease.

The mycobacteria possess a complex outer cell wall comprising an asymmetric lipid bilayer. The inner layer of the cell wall is a peptidoglycan layer that is linked via phosphodiester bond to the complex carbohydrates and arabinogalactan which are linked to high-molecular weight mycolic acids that forms a glycolipid [43]. Therefore, the complex waxy outer layer of *M. tuberculosis*, infection of host, survival in dormant form and reactivation in host to cause disease may be some of the reasons that require a variety of serine hydrolases in *M. tuberculosis*.

In conclusion, the present study has established that the PE-PPE domain from mycobacteria belongs to the family of serine α/β hydrolase proteins; biochemical characterization would establish the precise function of these proteins. Given the importance of serine hydrolases in the synthesis of waxy lipid rich cell wall in mycobacteria during dormancy and reactivation, studies on these proteins will contribute to the drug and vaccine design targeted towards TB.

## Supporting Information

Figure S1 **Multiple sequence alignment of the PE-PPE domain that encodes a serine hydrolase in** ***M. tuberculosis*** **strain H37Rv and some bacterial serine hydrolases of known structure generated using the program MAPSCI.** The conserved pentapeptide sequence motif is represented in box. The amino acid residues in the catalytic triad are represented by * and the residues in the oxyanion hole are represented by #. The PDB_ID and chain identity of the known crystal structures are provided.
(DOC)

Table S1 List of proteins comprising the PE-PPE domain in mycobacterial genomes. NCBI_IDs of the proteins are given.
(DOC)
