# Supplementary material for: The PE-PPE Domain in Mycobacterium Reveals a Serine α/β Hydrolase Fold and Function: An In-Silico Analysis
Source: PLoS One. 2011 Feb 10;6(2):e16745. doi: 10.1371/journal.pone.0016745 (PMC3037379; doi:10.1371/journal.pone.0016745)
Supplement: Figure S1 — Multiple sequence alignment of the PE-PPE domain that encodes a serine hydrolase in M. tuberculosis strain H37Rv and some bacterial serine hydrolases of known structure generated using the program MAPSCI. The conserved pentapeptide sequence motif is represented in box. The amino acid residues in the catalytic triad are represented by * and the residues in the oxyanion hole are represented by #. The PDB_ID and chain identity of the known crystal structures are provided. (DOC) [file pone.0016745.s001.doc]

Figure S1:

#

1CEX:A RTTRDDLINGNSASC--ADVIFIYARGSTETG--N----L----GTLGPSIASNLE-SAF

2CZQ:A --------ATSSAC---PQYVLINTRGTGEPQ--G----Q----SAGFRTMNSQITAA-L

3HC7:A -----------------SKPWLFTVHGTGQPD--P----L---GPGLPADTARDVL-D-I

1BS9:A -------------SC--PAIHVFGARETTASP--G----Y-----GSSSTVVNGVLSA-Y

3AJA:B ------------ADC--PDVMMVSIPGTWESSPTDDPFNPTQFPLSLMSNISKPLAEQ-F

Rv0160c -----------LAAE--SPITALIMGGTNNP---LPDPE------YVTDINKAFIQTL-F

Rv1800 ------------AAA--AQTVGLVMGGSGTPIPSA---RY--------VELANALYMS-G

Rv2608 ------------NGG--PGVTALVMGGTDSLL-PL----P-----NIPLLEYAARFIT-P

Rv3539 -----------------------TVPGASPVHAATLLPFIGRLLAARYAELNTAIGTN-W

Rv3822 -------------------DTALIVPGTAPS---P--YGPLRSLYHFNPAMQPQIGAN-Y

Rv0151c ------------PMF--NQNTAIIMGGTGSPIPTP---SY--------VNAITTLFID-P

Rv0152c --------AMIPPFP--ANLTTLFFGPTGIPL-PP----P--------SMLTPPIRCR-S

Rv0159c ------------LSG--NPLTALMMGGTGEP---ILSDR------VLAIIDSAYIRPI-F

Rv1184c --------------TAK---VVYALGGARMP---G--IPWYEYTNQAGSQYFPNAKHD-L

Rv1430 ------------LGS--GGRTALILGSTGTP---RPPFD------YMQQVYDRYIAPH-Y

110 120 130 140

1CEX:A GKD-GVWIQGVGGAYRATLG-------------------DNALPRG--TSSAAIREMLGL

2CZQ:A S---GGTIYNTV--YTADF--------------------SQ-N-----SA-AGTADIIRR

3HC7:A -----YRWQPIG-NYPAAAF-------------------PM-W---PSVE-KGVAELILQ

1BS9:A P-G--STAEAIN--YPACGG---------QSSCGG---ASY-S---SSVA-QGIAAVASA

3AJA:B G-PDRLQVYTTP--YTAQFH-N--P-FAADK---Q---MSY-N---DSRA-EGMRTTVKA

Rv0160c P-G--AVSQGLF--TPEQFW-PVTP-D--LG---N---LTF-N---QSVT-EGVALLNTA

Rv1800 S-VPGVIAQALF--TPQGLY-P--V-V-VIK---N---LTF-D---SSVA-QGAVILESA

Rv2608 V-HPGYTATFLE--TPSQFF-P--F-T-GLN---S---LTY-D---VSVA-QGVTNLHTA

Rv3539 F-P-GTTPEVVS--YPATIG-V--L-SGSLG---A---VDA-N---QSIA-IGQQMLHNE

Rv3822 Y-NPTATRHVVS--YPGSFW-P--V-T-GLN---S---PTV-G---SSVS-AGTNNLDAA

Rv0151c V-VSNPVVKALV--TPEELY-P--I-T-GVK---S---LPF-Q---TSVQ-LGLQILDGA

Rv0152c V-R--RALQAVF--TPEELY-P--L-T-GVR---S---LVL-N---TSVE-EGLTILHDA

Rv0159c G-PNNPVAQYT---P-EQWWP-------------FIGNLSL-D---QSIA-QGVTLLNNG

Rv1184c I-DYPAGAAFSW--WPTMLL-P--PGS-HQD---N---MTV-G---VAVK-DGTNSLDNA

Rv1430 L-G--YAFSGLY--TPAQFQ-P--W-T-GIP---S---LTY-D---QSVA-EGAGYLHTA

150 160 170 180

*#

1CEX:A FQQANTKCPDATLIAGGYSQGAALAAASIE-D-------------------LDSAI-RDK

2CZQ:A INSGLAANPNVCYILQGYSQGAAATVVALQ-Q------------LGT-SG----AA-FNA

3HC7:A IELKLDADPYADFAMAGYSQGAIVVGQVLKHH------------ILP-PTGRLHRF-LHR

1BS9:A VNSFNSQCPSTKIVLVGYSQGGEIMDVALC-GGGDPNQGYTNTAV-----QLS-SSAVNM

3AJA:B MTDMNDRCPLTSYVIAGFSQGAVIAGDIAS-D------------IGN-G-RGP-VD-EDL

Rv0160c VNNQLA--LDNKVVAFGYSQSATIINNYIN-S------------LMA-M-GSP-NP-D--

Rv1800 IRQQIA--AGNNVTVFGYSQSATISSLVMA-N------------LAA-S-ADP-PS-PDE

Rv2608 IMAQLA--AGNEVVVFGTSQSATIATFEMR-Y------------LQSLP-AHL-RP-GLD

Rv3539 ILAATA--SGQPVTVAGLSMGSMVIDRELA-Y------------LAI-D-PNA-PP-SSA

Rv3822 IRSTD-----GPIFVAGLSQGTLVLDREQA-R------------LAN-D-PTA-PP-PGQ

Rv0151c IWEQIN--AGNHVTVFGYSQSAVIASLEMQ-H------------LIS-L-GPN-AP-SPS

Rv0152c IMVELA-TTGNAVTVFGWSQSAIIASLEMQ-R------------FTA-M-GGA-AP-SAS

Rv0159c INAELQ--NGHDVVVFGYSQSAAVATNEIR-A------------LMALP-PGQ-AP-DPS

Rv1184c IHHGT-----DPAAAVGLSQGSLVLDQEQA-R------------LAN-D-PTA-PA-PDK

Rv1430 IMQQVA--AGNDVVVLGFSQGASVATLEMR-H------------LASLP-AGV-AP-SPD

190 200 210 220

1CEX:A IAGTVLFGYTKNLQNR-GR-----------------------------------------

2CZQ:A VKGVFLIGNPDH--KSGLTC-NVDSNG------GTTTRN-VNGLSV--A-----------

3HC7:A LKKVIFWGNPMR--QKG--F-AH---SDEWIHPV-AA-PDTLGIL---E-----------

1BS9:A VKAAIFMGDPMF--RAGLSY-EV----------G-TC-A-AGGFDQ--------------

3AJA:B VLGVTLIADGRR--QMGVGQ-DV---G------P-NP-A-GQGAEITLHE-VPALSALGL

Rv0160c DISFVMIGSGNN--PVGGLL-AR---F------P-GF-Y-IPFLDVPFNG-A------TP

Rv1800 L-SFTLIGNPNN--PNGGVA-TR---F------P-GI-S-FPSLGVTATG-A--------

Rv2608 ELSFTLTGNPNR--PDGGIL-TR---F------G-FS-I-PQLGFTLSGA-T--------

Rv3539 L-TFVELAGP----------------------------------ERGLAQ-TYLPVGTTI

Rv3822 L-TFIKAGDPNN--LLWRAF-RP---G------T-HV-P-IIDYTVPAPA-E--------

Rv0151c QLNFILIGNEMN--PNGGIL-AR---I------P-GL-N-VTTLGLPFYG-A--------

Rv0152c DLNFVLVGNEMN--PNGGML-AR---F------P-DL-T-LPTLDLTFYG-A-------T

Rv0159c RLAFTLIGNINN--PNGGVL-ER---Y------V-GL-Y-LPFLDMSFNG-A-------T

Rv1184c L-QFTTFGDPTG--RHAFGAS---------------------FLARIFPPGS--HIPIPF

Rv1430 QLSFVLLGNPNN--PNGGIL-AR---F------P-GL-Y-LQSLGLTFNG-A--------

230 240 250 260

*

1CEX:A ------------I-PNY--PADRTKVFCNTGDLVCTG--S--------------------

2CZQ:A -YQG--S-----VPSGW---VSKTLDVCAYGDGVCDT---AHGF----------------

3HC7:A ---D--R-----L-ENLEQYGFEVRDYAHDGDMYASI---K-EDDLHEYEVAIGRIVMKA

1BS9:A ------RPAGFS----C-PSAAKIKSYCDASDPYCCNGSN--------------------

3AJA:B TMTG-PR--PGG----FGALDNRTNQICGSGDLICSA---P---E--Q-AFS--------

Rv0160c ANSP--Y-----P----------THIYTAQYDGIAHA---P---Q--F-PLRI-------

Rv1800 TPHN--L-----Y---P------TKIYTIEYDGVADF---P---R--Y-PLNF-------

Rv2608 PADA--Y-----P----------TVDYAFQYDGVNDF---P---K--Y-PLNV-------

Rv3539 PIAG-YT--VGN----APESQYNTSVVYSQYDIWADP---P---D--R-PWN--------

Rv3822 -SQY--D-----T-----------INIVGQYDIFSDP---P---N--R-PGNL-------

Rv0151c -TPD--N-----P---Y-----PTTTYTLEYDGFADF---P---R--Y-PLNV-------

Rv0152c PSDT--I-----Y---P------TAIYTLEYDGFADF---S---R--Y-PLNF-------

Rv0159c PPDS--P-----Y---Q------TYMYTGQYDGYAHN---P---Q--Y-PLNI-------

Rv1184c IEYTMPQ--QVD----S---QYDTNHVVTAYDGFSDF---P---D--R-PDN--------

Rv1430 -TPD--T-----D---Y-----ATTIYTTQYDGFADF---P---K--Y-PLNI-------

270 280

*

1CEX:A -----------------------------------LI-V--A--------APHLAYG---

2CZQ:A -----------------------------------G--I--N--------AQHLSYPSD-

3HC7:A SGFIGGRDSVVAQLIELGQRPITEGIALAGAIIDA---LTFFARSRMGDKWPH-LY----

1BS9:A -----------------------------------------A--------ATHQGYGS--

3AJA:B ------------------------V-FNLPKTL-ETLSGSAA-------GPVHALYNTPQ

Rv0160c ------------------------L--SDINAF-MGY-F--Y---------VHNTYPELM

Rv1800 ------------------------V--STLNAI-AGT-Y--Y---------VHSNYFILT

Rv2608 ------------------------F--ATANAI-AGI-L--F---------LHSGLIALP

Rv3539 ------------------------L-LAGANAL-MGA-A--Y---------FHDLTAYAA

Rv3822 ------------------------L--ADLNAI-AAG-G--Y--------YGHSATAFSD

Rv0151c ------------------------L--SDINAV-FGI-L--T---------VHTTYADLT

Rv0152c ------------------------I--SDLNAV-AGI-T--F---------VHTKYLDLT

Rv0159c ------------------------L--SDLNAF-MGI-R--W---------VHNAYPFTA

Rv1184c ------------------------L-LAVANAA-IGA-A--I---------AHTPIGFTG

Rv1430 ------------------------L--ADVNAL-LGI-Y--Y---------SHSLYYGLT

290 300

1CEX:A ------------P----DARGPAPEFLIEKVRA--VRGS-----

2CZQ:A -----------QG----VQ-TMGYKFAVNKLGGSA---------

3HC7:A ------------N-----R-YPAVEFLRQ---------------

1BS9:A ------------E----YG-SQALAFVKSKL-G-----------

3AJA:B FWV-ENG----QT----AT-QWTLEWARNLVEN--AP--HP---

Rv0160c ATQ-VDN----AVPLPTSP-GYTGNTQYYMFLT--QD--LP---

Rv1800 PEQ-IDA----AV----PL-TNTVGPTMTQYYI--IR--TENLP

Rv2608 PDL-ASG----VV----QP-VSSPDVLTTYILL--PS--QDLP-

Rv3539 PQQ-GIE----IA----AV-TSSLGGTTTTYMI--PS--GYS--

Rv3822 PAR-VAPRDITTT----TN-SLGATTTTYFIRT--DQ--LP---

Rv0151c PAQ-IAS----AT----QL-PTQGTTSNTYYII--ET--EHLP-

Rv0152c PAQ-VEG----ATKLPTSP-GYTGVTDYYIIRT--EN--RP---

Rv0159c AEV-ANA----VP----LP-TSPGYTGNTHYYM--FL--TQDLP

Rv1184c PGDVPP-----QN----IR-TTVNSRGATTTTY--LV--PVN--

Rv1430 PEQ-VAS----GI----VL-PVSSPDTNTTYIL--LP--NED--

310 320 330
