# Supplementary material for: The PE-PPE Domain in Mycobacterium Reveals a Serine α/β Hydrolase Fold and Function: An In-Silico Analysis
Source: PLoS One. 2011 Feb 10;6(2):e16745. doi: 10.1371/journal.pone.0016745 (PMC3037379; doi:10.1371/journal.pone.0016745)
Supplement: Table S1 — List of proteins comprising the PE-PPE domain in mycobacterial genomes. NCBI_IDs of the proteins are given. (DOC) [file pone.0016745.s002.doc]

Table S1:

| Organism | Proteins with PE domain & PE-PPE domain | Proteins with PPE domain & PE-PPE domain | Hypothetical proteins with PE-PPE domain |
| --- | --- | --- | --- |
| *M. tuberculosis* CDC1551 | NP_334571.1  NP_335924.1  NP_334570.1  NP_334576.1  NP_334577.1 | NP_337185.1  NP_336306.1  NP_338188.1 | NP_338483.1  NP_335664.1 |
| *M. tuberculosis* H37Rv | YP_177696.1  YP_177810.1  YP_177695.1  YP_177697.1  YP_177698.1 | YP_177893.1  YP_177839.1  YP_177987.1 | NP_218339.1  NP_215700.1 |
| *M. tuberculosis* H37Ra | YP_001282744.1  YP_001281438.1  YP_001281446.1  ZP_02552557.1  YP_001281447.1 | YP_001283971.1  YP_001283129.1  YP_001284925.1 | ZP_02548944.1  YP_001281439.1  ZP_02552547.1  ZP_02552214.1  YP_001285213.1  YP_001282494.1 |
| *M. bovis* AF2122/97 | NP_853823.1  NP_855117.1  NP_853822.1  NP_853831.1  NP_853830.1 | NP_855481.1  NP_856286.1  NP_857208.1 | NP_857489.1  NP_854870.1 |
| *M. bovis* BCG str. Pasteur 1173P2 | YP_977583.1  YP_976286.1  YP_976295.1  YP_976294.1 | YP_977924.1  YP_978719.1  YP_979682.1 | YP_976287.1  YP_979964.1  YP_977338.1 |
| *M. bovis* BCG str. Tokyo 172 | YP_002643224.1  YP_002644522.1  YP_002643223.1  YP_002643232.1  YP_002643231.1 | YP_002644872.1  YP_002645676.1  YP_002646644.1 | YP_002646925.1  YP_002644275.1 |
| *M. kansasii*  ATCC 12478 | ZP_04750460  ZP_04746832  ZP_04746828  ZP_04746825  ZP_04746830  ZP_04746817  ZP_04746816  ZP_04746831  ZP_04751910  ZP_04746829  ZP_04746815  ZP_04750329 | ZP_04751918  ZP_04751917  ZP_04747254 |  |
| *M. marinum* M | YP_001850539  YP_001848693  YP_001848692  YP_001848694  YP_001851707  YP_001848706  YP_001852582 YP_001852247  YP_001848705  YP_001848691  YP_001851742  YP_001848704  YP_001853198 | YP_001849339  YP_001849803 |  |
| *M. ulcerans* Agy99 | YP_905107.1  YP_905059.1 | YP_906263.1 |  |
| *M. parascrofulaceum*  ATCC BAA-614 |  |  | ZP_06848426.1 ZP_06852104.1 ZP_06849370.1 |
| *M. smegmatis* str. MC2 155 |  |  | YP_889694.1 YP_887070.1 YP_888993.1 YP_890365.1 YP_884825.1 YP_889988.1 YP_889594.1  YP_885568.1 |
| Mycobacterium sp. JLS |  |  | YP_001069995.1 YP_001068542.1 YP_001069270.1 YP_001072916.1 YP_001068711.1 YP_001068707.1 YP_001073586.1 YP_001071123.1 YP_001072947.1 YP_001072948.1 |
| Mycobacterium sp. KMS |  |  | YP_937773.1 YP_936971.1 YP_936265.1 YP_940348.1 YP_941018.1 YP_938853.1 YP_936435.1 YP_936436.1 YP_936431.1 YP_940378.1 YP_940379.1 |
| Mycobacterium sp. MCS |  |  | YP_638900.1 YP_638120.1 YP_637425.1 YP_641442.1 YP_642111.1 YP_639988.1 YP_637596.1 YP_637597.1 YP_637592.1 YP_641472.1  YP_641473.1 |
| *M. vanbaalenii* PYR-1 |  |  | YP_952665.1 YP_953911.1 YP_953352.1 YP_955506.1 YP_952079.1 YP_951131.1 YP_953914.1 YP_953918.1 YP_953778.1 YP_955594.1 YP_953910.1 YP_951605.1 YP_950898.1 YP_951855.1 YP_952889.1 YP_955503.1 YP_950878.1 YP_956151.1  YP_955503.1  YP_950878.1  YP_952432.1 YP_954864.1 |
| *M. abscessus* ATCC  19977 |  |  | YP_001701682.1  YP_001704868.1 |
| *M. avium* 104 |  |  | YP_880985.1 |
| *M. leprae* TN |  |  | NP_301893.1 |
| *M. leprae* Br4923 |  |  | YP_002503523.1 |
